# Gut Microbiota Combined With Metabolomics Reveals the Repeated Dose Oral Toxicity of β-Cyclodextrin in Mice

**DOI:** 10.3389/fphar.2020.574607

**Published:** 2021-01-14

**Authors:** Shuangyu Lv, Xiaomei Zhang, Yu Feng, Qiying Jiang, Chenguang Niu, Yanjie Yang, Xinchun Wang

**Affiliations:** ^1^ Institute of Molecular Medicine, School of Basic Medical Sciences, Henan University, Kaifeng, China; ^2^ Key Laboratory of Clinical Resources Translation, The First Affiliated Hospital of Henan University, Kaifeng, China

**Keywords:** β-cyclodextrin, gut microbiota, metabolomics, oral exposure, mice

## Abstract

Βeta-cyclodextrin (β-CD) with a hydrophobic cavity enables the formation of inclusion complexes with organic molecules. The formation of host–guest complexes makes the application of β-CD popular in many fields, but their interaction with organisms is poorly understood. In the present study, the effect of β-CD on gut microbiota (16S rRNA gene sequencing), serum metabolites (gas chromatography–mass spectrometry platform), and their correlation (Pearson correlation analysis) was investigated after 14 days repeated oral exposure in mice. β-CD did not significantly affect the α-diversity indexes, including Richness, Chao1, Shannon and Simpson indexes, but disturbed the structure of the gut bacteria according to the result of principal component analysis (PCA). After taxonomic assignment, 1 in 27 phyla, 2 in 48 classes, 3 in 107 orders, 6 in 192 families, and 8 in 332 genera were significantly different between control and β-CD treated groups. The serum metabolites were significantly changed after β-CD treatment according to the result of unsupervized PCA and supervised partial least squares-discriminant analysis (PLS-DA). A total of 112 differential metabolites (89 downregulated and 23 upregulated) were identified based on the VIP >1 from orthogonal PLS-DA and *p* <0.05 from Student’s *t*-test. The metabolic pathways, including ABC transporters, pyrimidine metabolism, purine metabolism, glucagon signaling pathway, insulin signaling pathway, and glycolysis/gluconeogenesis, were enriched by KEGG pathway analysis. Our study provides a general observation of gut microbiota, serum metabolites and their correlation after exposure to β-CD in mice, which will be helpful for future research and application of β-CD.

## Introduction

Βeta-cyclodextrin (β-CD) is a cyclic oligosaccharide with a truncated cone shape. β-CD has a hydrophobic cavity and hydrophilic outer edge (Wu J.-W. et al., 2020; Zhong et al., 2020). The hydrophobicity of β-CD cavities enables formation of inclusion complexes with organic molecules by noncovalent intermolecular interactions, such as electrostatic, hydrophobic and Van der Waals interactions (Bouhadiba et al., 2020). The formation of host–guest complexes is important for their application (Bouhadiba, et al., 2020), including pharmaceuticals (Mura 2020), attachment and degradation of contaminants (Wu, et al., 2020; Zhou et al., 2020), and nanosponge preparation (Pawar et al., 2019). β-CD can reduce the agglomeration of nanomaterials and increase their specific surface area (Zhou, et al., 2020). The application and inevitable release of β-CD increase the opportunity for exposure, which may be harmful to humans and other organisms. However, the interaction between β-CD and organisms is poorly understood.

After oral exposure, β-CD passes through the digestive tract, where it may interact with the gut microbiota in the luminal environment. The gut microbiota is composed of different types of bacteria, viruses and fungi, and comprises 10-fold more cells than the human body (Yang et al., 2020; Zhang L. et al., 2020). The composition of gut microbiota plays crucial roles in the maintenance of host health (Chen et al., 2019; Yue et al., 2019). Dysbiosis of the gut microbiome can affect energy metabolism, nutritional digestion and absorption, immune status, and occurrence of many diseases (e.g. obesity, diabetes, arteriosclerosis, enteritis and hepatitis) (Chen et al., 2019; Vojinovic et al., 2019; Yue et al., 2019; Luo et al., 2020). The composition and diversity of the gut microbial community is highly sensitive to exogenous stressors (Chen et al., 2019), but the influence of β-CD on the gut microbiota is little known.

Symbiotic bacteria can regulate host metabolism, and circulating metabolite levels often act as an intermediary between the gut microbiota and host biology (Luo et al., 2020; Wu W. et al., 2020). Therefore, we speculate that oral exposure to β-CD may affect the gut microbiota and then alter serum metabolism. The metabolome as the final downstream product of the genome, transcriptome, and proteome, provides global and system-wide metabolic changes (Lindeque et al., 2018; Yang et al., 2019). Gas chromatography–mass spectrometry (GC–MS) with higher sensitivity and resolution is a robust platform for analysis of small molecules, and has been widely applied in metabolomics studies (Duan et al., 2020). We selected the GC–MS platform for detecting serum metabolites in this study. The combination of metabolome and microbiome is a promising approach to evaluate the relationship between host metabolism and gut microbiota (Luo et al., 2020).

The aim of this study was to explore whether oral administration of β-CD affected the composition and diversity of the gut microbiota in mice, and then indirectly caused a series of changes in circulating metabolites. We integrated gut microbiota, serum metabolites, and their correlation to explore the possible effects of β-CD on host health.

## Materials and Methods

### Animals and Treatment

Male Kunming mice of 22 ± 2 g (age 6–8 weeks) were supplied by the Animal Center of Henan Province (Zhengzhou, China). The mice were housed (4 or 5/cage) in a controlled environment of 22 ± 1 °C, 50–60% relative humidity, and 12 h light–dark cycle, with free access to food and water. The study and protocol were approved by the Committee of Medical Ethics and Welfare for Experimental Animals of Henan University School of Medicine (HUSOM2020-257).

Mice were randomly divided into the control group (normal saline) and β-CD treated (#C8510; Solarbio Science and Technology Co. Ltd., Beijing, China) group, with 10 animals per group. Mice were gavaged consecutively for 14 days with a total volume of 100 μL (17.5 g/L β-CD). The dose was selected based on previous studies of Jung et al. research on microbial communities and the aqueous solubility of 1.85 g/100 ml (Yu et al., 2008; Jung et al., 2015). At 2 h after the last gavage, fresh feces were collected, snap-frozen in liquid nitrogen, and stored at −80 °C for subsequent analysis. The body weight was measured, and blood was collected before killing by cervical dislocation.

### Coefficients of Organs

After the mice were sacrificed, heart, liver, spleen, lung, and kidney tissues were carefully removed and weighed. The coefficients of organs were calculated as the ratio of organs weight (mg) to body weight (g).

### Gut Microbiota Analysis

Gut microbiota were analyzed by 16S rRNA gene sequencing. Details on DNA extraction, 16S rRNA gene sequencing, sequence processing and analysis are shown below.

*DNA Extraction and 16S rRNA Gene Sequencing* Genomic DNA was extracted using a QIAamp 96 PowerFecal QIAcube HT kit (Hilden, Germany). DNA quantity and quality were determined with a NanoDrop 2000 UV-Vis Spectrophotometer (Thermo Scientific, Wilmington, DE, United States) and agarose gel electrophoresis. DNA samples were diluted to 1 ng/μL and used as template for PCR amplification of bacterial 16S rRNA genes. Universal primers 343F (5′-TACGGRAGGCAGCAG-3′) and 798R (5′-AGG​GTA​TCT​AAT​CCT-3′) for V3–V4 variable regions were used. Amplicon quality was detected by gel electrophoresis. After purification with Agencourt AMPure XP beads, another round of PCR was done. The final purified amplicon was quantified by Life Technologies Qubit dsDNA assay kit (Carlsbad, CA, United States). Equal amounts of purified amplicon were pooled, and sequencing was carried out using an Illumina MiSeq platform (San Diego, CA, United States).

*Sequence Processing and Analysis* Raw sequences data were preprocessed using Trimmomatic software (Bolger et al., 2014) to cut off ambiguous bases (N) and low quality sequences with average quality scores below 20. FLASH software was used to assemble the paired-end reads following the parameters of 10 bp of minimal overlapping, 200 bp of maximum overlapping, and 20% maximum mismatch rate (Reyon et al., 2012). Further denoizing was performed using QIIME software (version 1.8.0), and we abandoned the sequences with ambiguous, homologous sequences, below 200 bp or chimeras (Caporaso et al., 2010). Removed primer sequences and clean reads were clustered to generate operational taxonomic units (OTUs) based on 97% similarity cutoff by Vsearch software (version 2.4.2) (Edgar et al., 2011). The representative read (maximum richness) of each OTU was selected using the QIIME package, and then blasted and annotated against Silva database (version 123) using RDP classifier to obtain taxonomic information (Wang et al., 2007). The confidence threshold was set to 70%. The community compositions of each sample were counted at the level of phylum, class, order, family, and genus. The α-diversity was calculated for each sample, including Richness, Chao1, Simpson, and Shannon indexes. Principal component analysis (PCA) was used to reveal the β diversity. Analysis of variance (ANOVA) was used to compare the species differences between the control and β-CD treated groups.

### Metabolomics Research

Serum metabolites were analyzed by GC–MS, and detailed information of sample preparation, data preprocessing, and GC–MS and multivariate analyses are shown below.

*Sample Preparation* Serum was obtained by centrifugation at 3,000 rpm for 10 min, followed by further centrifugation (Eppendorf 5810R, Germany) 12,000 rpm for 10 min at 4 °C. The supernatant was collected and stored at −80 °C. Thawed serum (80 μL) and 10 μL 0.3 mg/ml methanol solution of 2-chloro-l-phenylalanine (internal standard) were mixed and vortexed for 10 s. We added 240 μL cold mixture of methanol and acetonitrile (2/1, v/v) and vortexed for 1 min, then ultrasonicated in ice water for 5 min, and stored at −20 °C for 10 min. Samples were centrifuged at 12,000 rpm for 10 min at 4 °C, and 150 μL supernatant was collected and dried under vacuum. Subsequently, we added 80 μL 15 mg/ml methoxyamine hydrochloride in pyridine, vortexed vigorously for 2 min, and incubated at 37 °C for 90 min. We added 80 μL N,O-bis(trimethylsilyl)trifluoroacetamide (BSTFA, with 1% trimethylchlorosilane), 20 μL n-hexane and another 11 fatty acid methyl ester (C8/C9/C10/C12/C14/C16, 0.8 mg/ml; C18/C20/C22/C24/C26, 0.4 mg/ml) internal standard in chloroform, and derivatized at 70 °C for 1 h before GC–MS analysis. Quality control (QC) samples were prepared by mixing aliquots of all the samples and analyzing with the same procedure.

*GC–MS Analysis* The derivatized samples were analyzed using an Agilent 7890B GC system coupled to an Agilent 5977A MSD system (Agilent Technologies Inc., Santa Clara, CA, United States). We used a DB-5MS fused-silica capillary column (30 m × 0.25 mm × 0.25 μm; Agilent J and W Scientific, Folsom, CA, United States). Helium (>99.999%) was used as the carrier gas at a constant flow rate of 1.0 ml/min through the column. The temperature of the injector was maintained at 260 °C, and injection volume was 1 μL by splitless mode. The initial oven temperature was 60 °C, increased to 125 °C at a rate of 8 °C/min, to 210 °C at 5 °C/min, to 270 °C at 10 °C/min, to 305 °C at 20 °C/min and finally held at 305 °C for 5 min. The temperatures of the MS quadrupole and electron impact ion source were set to 150 and 230 °C, respectively. The collision energy was 70 eV. MS data were acquired in a full-scan mode (m/z 50–500), The time of solvent delay was set to 5 min. QC samples were detected at regular intervals (every 10 samples) throughout the analysis.

*Data Preprocessing and Multivariate Statistical Analysis* Raw data (.D format) were converted to.abf format by Analysis base File Converter software. MS-DIAL software was used to analyze the.abf data. Metabolites were annotated through Untarget database of GC–MS from Luming Biotechnology Co. Ltd. (Shanghai, China). All peak areas were processed by normalization of multi-interior label (RSD <0.3) according to the retention time partition period. Data were imported into R ropls package, where unsupervized PCA, supervised partial least squares-discriminant analysis (PLS-DA) and orthogonal PLS-DA (OPLS-DA) were performed. The differential metabolites were selected based on variable influence on projection (VIP) values of OPLS-DA model larger than 1.0, as well as *p* values of two-tailed Student’s *t*-test <0.05. Enrichment analysis of differential metabolites was performed by KEGG (http://www.kegg.jp/), and *p* < 0.05 was considered significantly enriched.

### Integration Analysis Between the Modified Gut Microbiota and Metabolites

All the differential phyla and genera according to ANOVA (*p* < 0.05), and significantly changed metabolites (VIP >1 and *p* < 0.05) were selected. The Pearson correlation coefficient between bacteria and metabolites was calculated; *p < 0.05 and **p < 0.01 according to Pearson correlation.

## Results

### Coefficients of Organs

No abnormal behavior including eating, drinking, or activity, was found in the β-CD treated group. The body weights of the β-CD treated group were comparable to those of the control group (22.79 ± 0.76 g to 31.99 ± 0.72 g) (Supplementary Figure S1). The coefficients of lung (control, 6.82 ± 0.21 mg/g), liver (control, 52.93 ± 1.67 mg/g), spleen (control, 2.63 ± 0.23 mg/g), heart (control, 5.42 ± 0.23 mg/g), and kidney (control, 13.78 ± 0.34 mg/g) are shown in Supplementary Table S1. None of them was affected by oral administration of β-CD for 14 days.

### Effects of Oral Exposure to β-CD on Gut Microbiota

**FIGURE 1:**
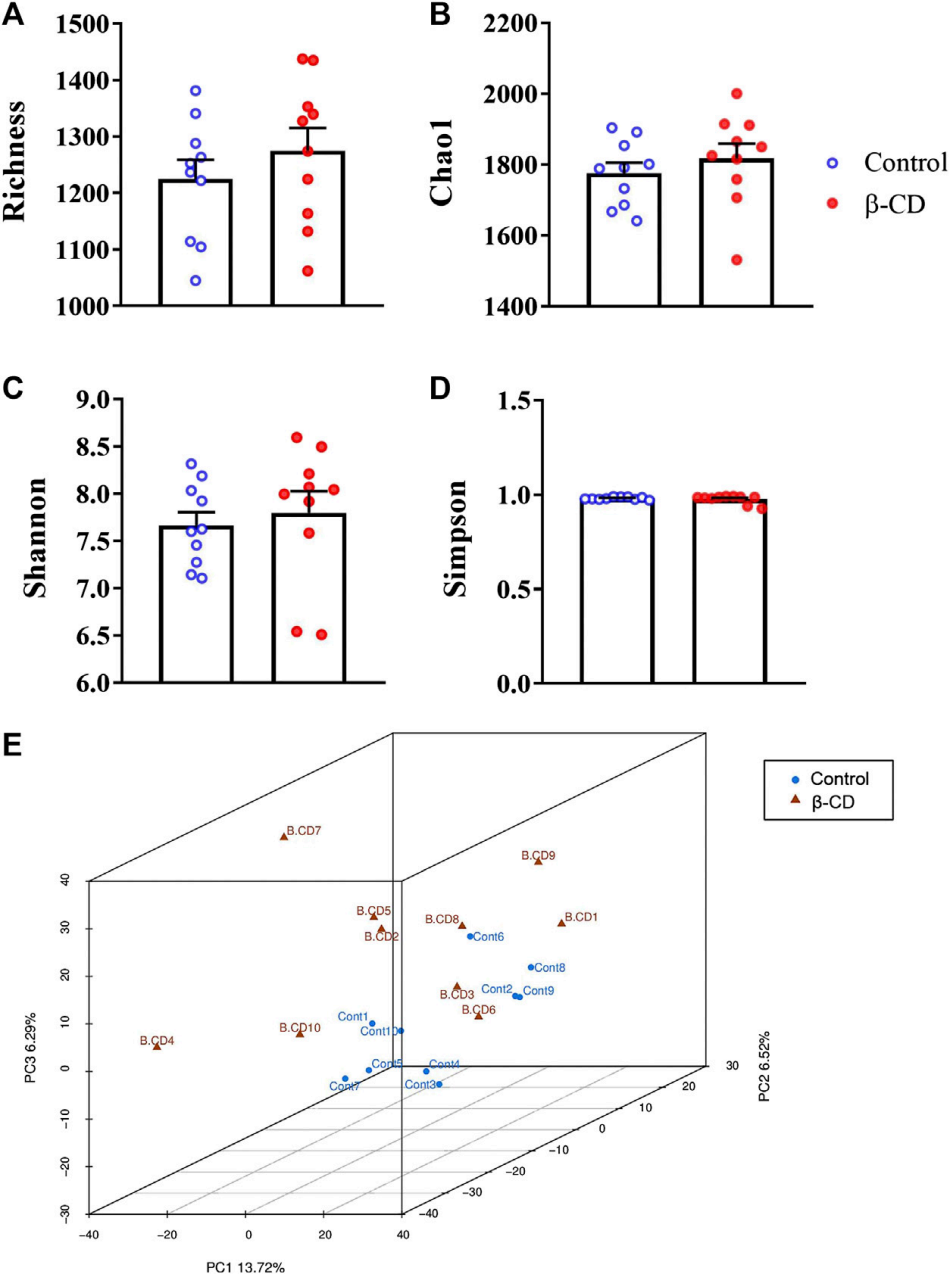
Alpha- and beta-diversity of the gut microbiota in mice after oral exposure to β-CD for 14 days. **(A)** Richness, **(B)** Chao1, **(C)** Shannon, **(D)** Simpson, **(E)** Principal component analysis (PCA). All data are presented as mean ± SEM (*n* = 10). *p* value vs. control according to Kruskal–Wallis. No significant difference was found between control and β-CDtreated groups.

Fresh fecal samples were collected after oral administration of β-CD for 14 days 16S rRNA gene sequencing was performed to construct the microbial community profiles. There were 17,004–33,139 valid tags in the fecal samples (Supplementary Table S2). A total of 3,031 OTUs were generated based on 97% similarity, including 1,064–1,463 OTUs per sample. The α-diversity indexes, including Richness, Chao1, Shannon, and Simpson indexes, were calculated (Figures 1A–D). None of the above indexes was disturbed by repeated oral administration of β-CD. β-CD affected the structure of the gut microbiota according to the result of PCA analysis (Figure 1E).

**FIGURE 2:**
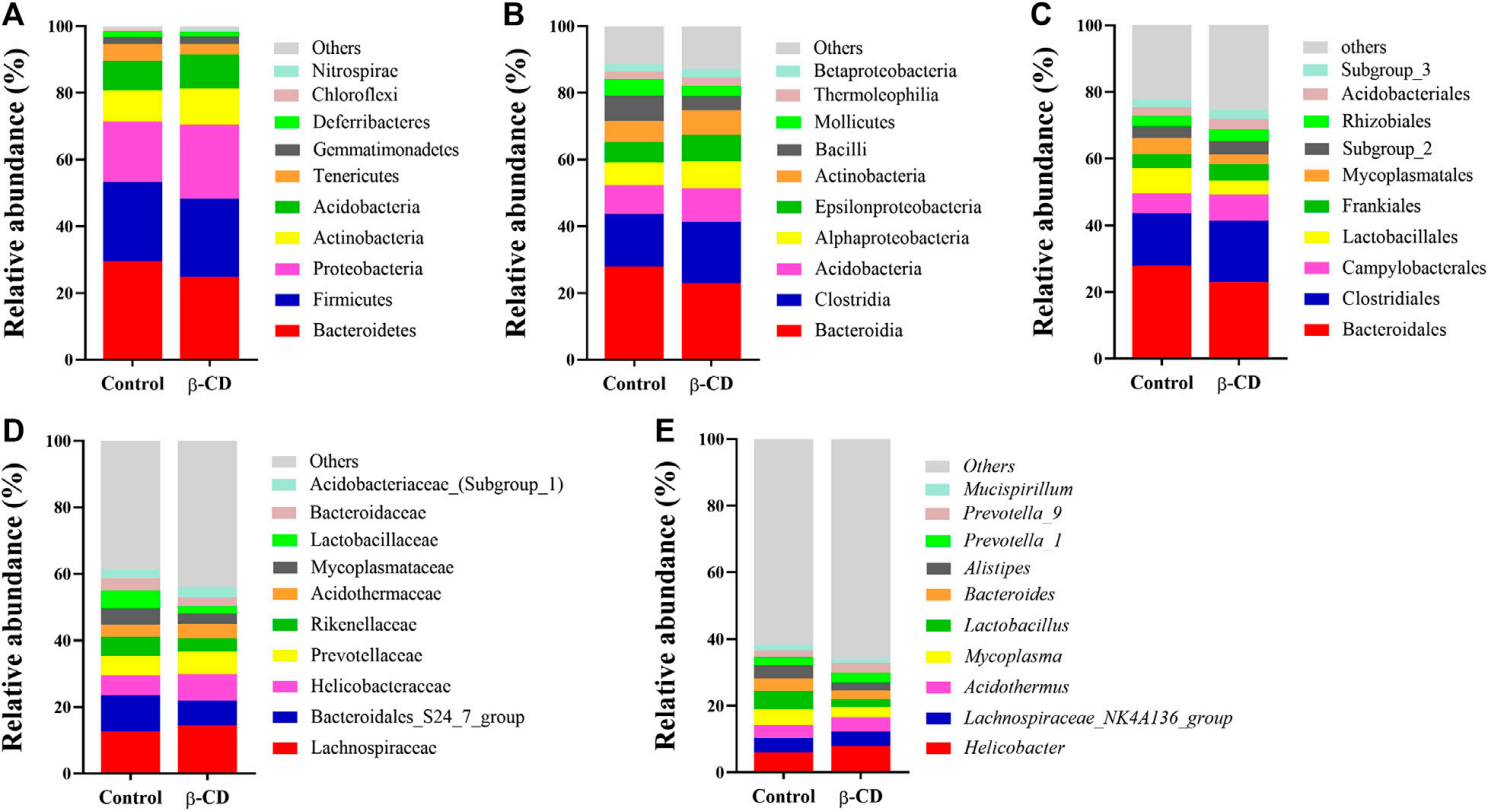
Taxonomic distributions of bacteria (Top 10) at the **(A)** Phylum, **(B)** Class, **(C)** Order, **(D)** Family, and **(E)** Genus.

**FIGURE 3:**
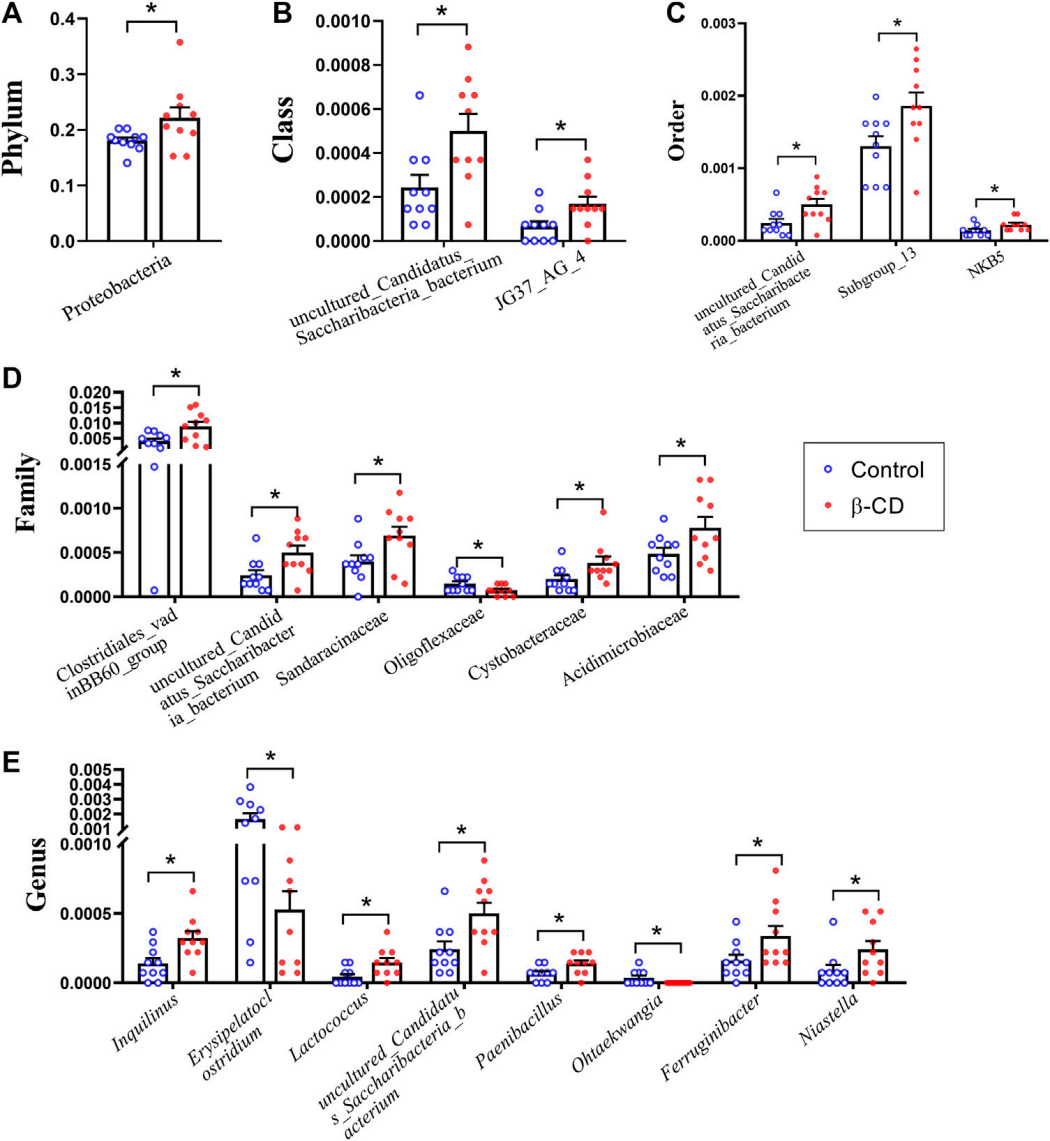
Scatter plots of significantly altered bacterial taxa, including **(A)** Phylum, **(B)** Class, **(C)** Order, **(D)** Family, **(E)** Genus by repeated oral exposure to β-CD for 14 days *p < 0.05 and **p < 0.01 according to ANOVA (*n* = 10).

According to the taxonomic assignment, the top 10 relative abundances of bacteria at the levels of phylum, class, order, family, and genus are shown in Figures 2A–E. At the phylum level, ∼90% of the sequences were within the top five phyla of *Bacteroidetes*, *Firmicutes*, *Proteobacteria*, *Actinobacteria* and *Acidobacteria*. The abundance of *Proteobacteria* was significantly increased in the β-CD treated group (*p* < 0.05, Figure 3A). The classification of bacteria tended to be dispersed in other levels, and 2 in 48 classes (*uncultured Candidatus Saccharibacteria bacterium*, *JG37 AG 4*), 3 in 107 orders (*uncultured Candidatus Saccharibacteria bacterium*, *Subgroup 13*, *NKB5*), 6 in 192 families (*Clostridiales vadinBB60 group*, *uncultured Candidatus Saccharibacteria bacterium*, *Sandaracinaceae*, *Oligoflexaceae*, *Cystobacteraceae*, *Acidimicrobiaceae*), and 8 in 332 genera (*Inquilinus*, *Erysipelatoclostridium*, *Lactococcus*, *uncultured Candidatus Saccharibacteria bacterium*, *Paenibacillus*, *Ohtaekwangia*, *Ferruginibacter*, *Niastella*) differed significantly between the control and β-CD treated group (Figures 3B–E). Taxonomic distributions and significant differences of bacteria at the species level were shown in Supplementary Figure S2.

### Effects of Oral Exposure to β-CD on Serum Metabolites

**FIGURE 4:**
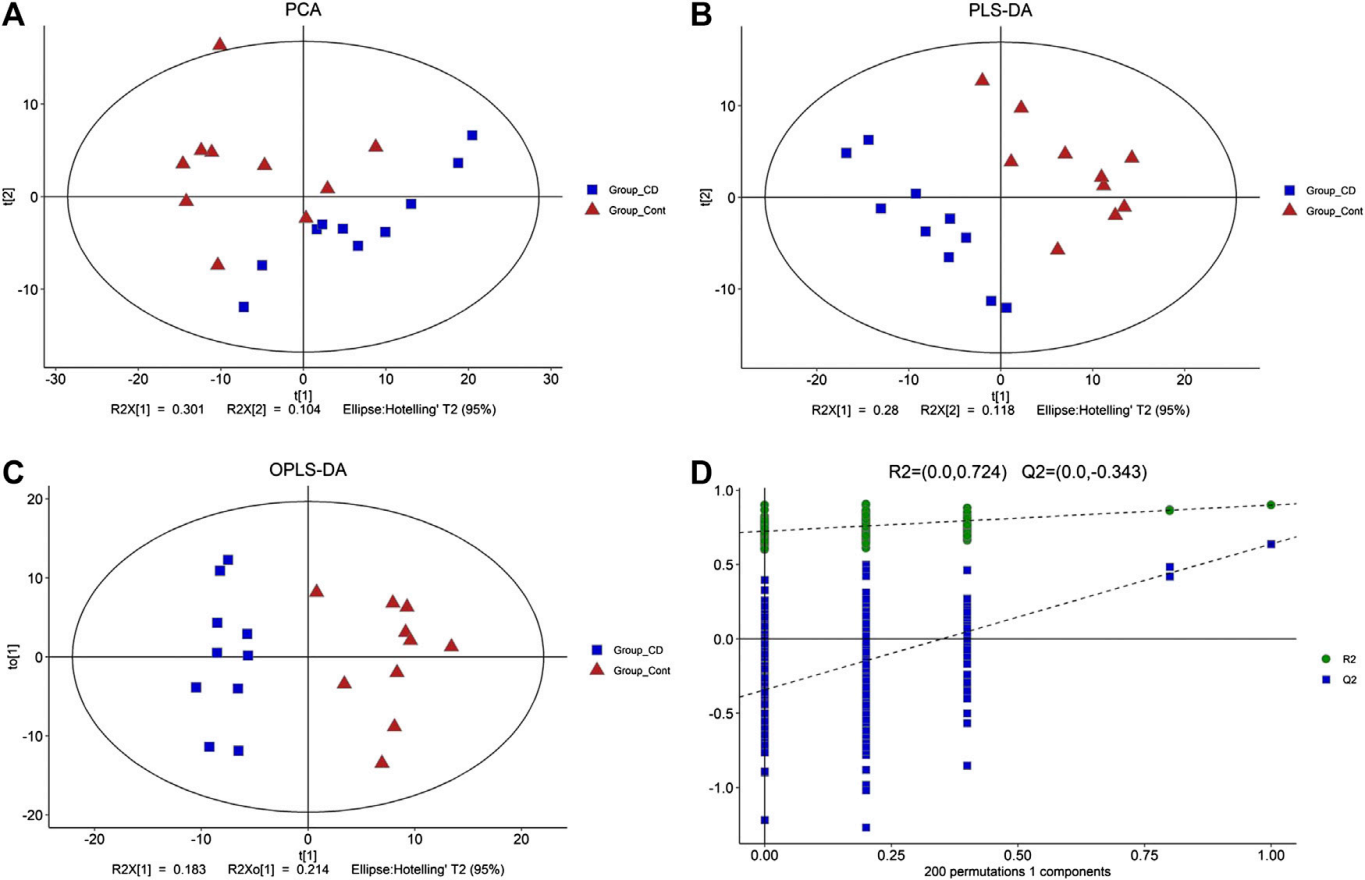
Metabolic profiling between control and β-CD treated group. **(A)** score plots of the PCA model, **(B)** score plots of the PLS-DA model, **(C)** scores plot of the OPLS-DA model, **(D)** plot of the permutation test (200 times) of the PLS-DA model.

Total ion current (TIC) chromatographs of control and β-CD treated samples are shown in Supplementary Figure S3. The reproducibility of methods was favorable according to the QC samples in PCA score plots (Supplementary Figure S4). The major spectrum can be assigned to specific metabolites by matching with the Untarget database of GC–MS from Lumingbio. Unsupervized PCA and supervised PLS-DA were selected to analyze the metabolic differences between the control and β-CD treated groups. There was an obvious separation tendency in PCA score plots (Figure 4A), and better separation was obtained in the PLS-DA model (Figure 4B), suggesting a significant difference after oral administration of β-CD for 14 days. Permutation test (200 times) was used to validate the reliability of the PLS-DA model (Figure 4D). OPLS-DA was performed and shown in Figure 4C.

**FIGURE 5:**
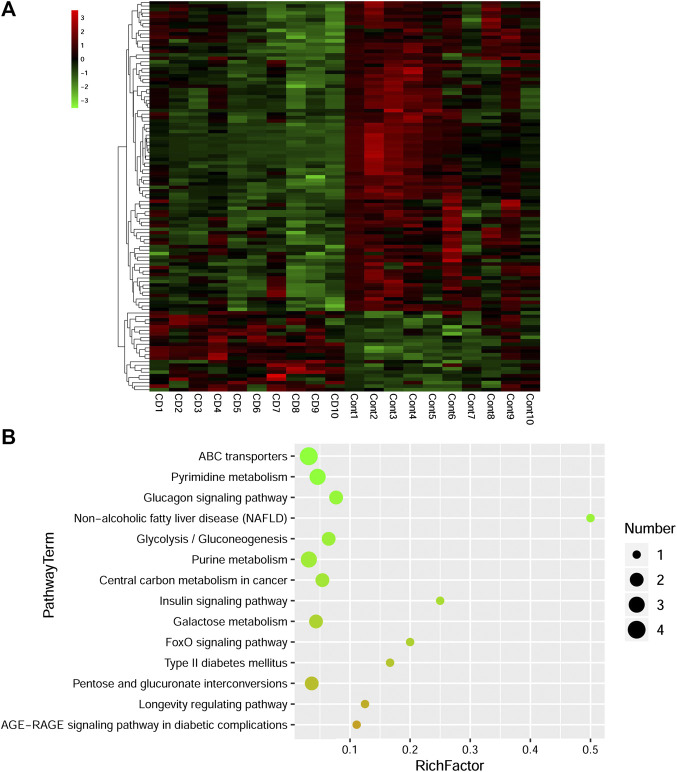
Heat map **(A)** and scatterplot of significantly enriched KEGG pathways **(B)** of differential metabolites induced by repeated oral administration of β-CD for 14 days. In the heat map, the *x* axis represents the sample name and the *y* axis represents the differential metabolite. Green color indicates a low abundance of metabolites and red color indicates a high abundance. In the scatterplot, the size of the dots represents the metabolites number.

Combined with VIP > 1 from OPLS-DA and *p* < 0.05 from two-tailed Student’s t-test, 112 differential metabolites (89 downregulated and 23 upregulated) were identified (Table 1). To show the differences in expression of metabolites, cluster analysis of all the changed metabolites was conducted (Figure 5A). The abscissa represents the sample names, and the ordinate represents the differential metabolites. The color from green to red indicates expression abundance of metabolites from low to high. KEGG pathway analyses were performed on 112 differential metabolites to find out the metabolic pathway regulated by β-CD. The top 10 significantly enriched pathways (*p* < 0.05) were ATP-binding cassette (ABC) transporters, pyrimidine metabolism, glucagon signaling pathway, nonalcoholic fatty liver disease (NAFLD), glycolysis/gluconeogenesis, purine metabolism, central carbon metabolism in cancer, insulin signaling pathway, galactose metabolism, and FoxO signaling pathway (Figure 5B).

**Table 1:** Significantly changed metabolites found in GC/MS-based metabolomic profiling.

Metabolites	Average Rt(min)	Quant mass	VIP	*p*-value	log_2_(FC)
(e)-9-tetradecenoic acid	22.43	283	1.06	4.95*E* − 02	0.48
1,2,3-trihydroxybenzene	17.44	342	2.14	4.16*E* − 05	−0.90
1,2-dihydroxycylohexane	7.07	258	1.19	4.07*E* − 02	−0.11
2,4-diaminobutyric acid	12.89	227	1.48	6.54*E* − 04	−0.21
2-deoxypentitol	15.81	211	1.38	1.89*E* − 03	−0.30
2-deoxypentonic acid	18.75	214	1.59	1.66*E* − 03	−0.67
2-hydroxybutanoic acid	6.87	131	1.60	1.44*E* − 03	−0.32
3,4-dihydroxyhydrocinnamic acid	24.50	256	1.14	3.87*E* − 02	0.73
3,6-anhydro-d-galactose	10.82	231	2.11	4.80*E* − 05	−0.93
3-arylcarbonyl-alanine	8.16	98	1.32	1.57*E* − 02	−0.33
3-deoxyhexitol	17.67	231	1.08	1.26*E* − 02	−0.33
3-desoxy-pentitol	21.94	231	1.13	2.36*E* − 02	−0.46
3-hydroxybenzoic acid	18.03	267	1.96	2.43*E* − 04	−0.51
3-pyridinol	6.45	152	1.21	2.02*E* − 02	−0.12
4',5-dihydroxy-7-glucosyloxyflavanone	25.20	443	1.89	4.61*E* − 05	−0.60
4-deoxyerythronic acid	11.92	292	1.05	4.28*E* − 02	−0.21
5-aminovaleric acid	13.16	154	1.92	1.40*E* − 04	−0.60
6-deoxyglucose	14.48	232	1.05	2.95*E* − 02	−0.55
Adenine	22.93	264	1.51	8.17*E* − 03	−0.56
Alkane	15.10	85	1.29	1.32*E* − 02	0.05
Allantoic acid	26.88	259	1.49	1.45*E* − 02	0.47
Alpha tocopherol	31.61	355	1.98	3.55*E* − 05	−1.10
Alpha-aminoadipic acid	19.96	260	1.42	4.09*E* − 02	0.63
Arabinofuranose	17.72	217	1.32	1.46*E* − 02	−0.41
Asparagine	15.28	115	1.13	1.82*E* − 02	−0.36
Beta-gentiobiose	13.13	180	1.01	2.79*E* − 02	0.64
Beta-glycerophosphoric acid	20.14	299	1.32	5.26*E* − 03	−0.23
Beta-hydroxymyristic acid	14.65	247	1.05	1.98*E* − 02	−0.78
Bisphenol a	27.35	357	1.21	2.24*E* − 02	0.84
Bisphosphoglycerol	16.77	299	1.06	2.34*E* − 02	−0.19
Catechin	31.14	295	1.90	2.62*E* − 05	−0.55
Catechol	11.39	254	1.22	2.20*E* − 02	−0.45
Chlorphentermine	11.47	313	1.40	1.07*E* − 02	0.24
Cholic acid	28.90	338	1.78	4.17*E* − 04	−0.77
Cyanoalanine	12.48	141	1.61	1.11*E* − 03	−0.52
Cytidine	16.69	267	1.82	7.14*E* − 04	−0.44
D-erythro-sphingosine	12.47	204	1.23	4.22*E* − 02	0.26
Digalacturonic acid	20.40	217	1.28	1.43*E* − 02	−0.42
Diglycerol	15.45	268	1.97	1.27*E* − 04	−0.60
D-lactic acid	6.80	117	1.08	2.67*E* − 02	−0.21
Dodecanol	13.93	243	2.17	6.85*E* − 05	−1.49
D-xylitol	20.06	217	1.50	4.24*E* − 03	−0.37
Eicosapentaenoic acid	30.73	91	1.09	4.28*E* − 02	−0.31
Enolpyruvate	7.24	147	1.93	9.84*E* − 05	−0.57
Ethanol phosphate	8.94	211	1.61	9.22*E* − 04	−0.28
Fructose	19.27	306	1.37	1.11*E* − 02	−0.42
Fructose-1,6-bisphosphate	28.14	299	1.49	8.25*E* − 03	0.51
Furoylglycine	9.61	169	2.07	1.73*E* − 04	−0.84
Galactaric acid	25.61	333	1.43	1.92*E* − 02	−0.62
Gamma-aminobutyric acid	9.35	102	1.92	1.58*E* − 05	−0.38
Glucose	15.41	197	1.49	5.80*E* − 05	−0.87
Glucose-1-phosphate	10.07	217	1.69	5.44*E* − 03	−0.40
Glycerol 3-phosphate	20.90	357	1.56	2.80*E* − 03	−0.35
Glycerol-alpha-phosphate	20.88	300	1.44	1.71*E* − 02	−0.17
Glycyl proline-1	23.21	373	1.79	4.77*E* − 05	−0.40
Guanidinosuccinate	16.75	328	1.74	6.40*E* − 04	−0.37
Heptadecanoic acid	27.94	327	1.44	1.54*E* − 02	−0.28
Hexadecane-1,2-diol	28.69	299	1.50	1.31*E* − 03	−0.33
Hexadecanedioic acid	25.21	415	2.10	2.04*E* − 05	−1.07
Histidine	13.16	154	1.80	5.36*E* − 04	−0.51
Hydroxylamine	7.75	249	1.52	3.41*E* − 05	−0.70
Hypoxanthine	21.72	265	1.97	1.45*E* − 04	−1.45
Inositol-4-monophosphate	30.95	318	1.58	3.41*E* − 03	−0.38
Isochlorogenic acid	19.22	363	1.27	3.53*E* − 02	0.54
Kynurenic acid	12.43	304	1.72	1.02*E* − 03	−0.64
L-aspartic acid	15.73	232	1.46	6.95*E* − 03	−0.56
L-glutamine	21.12	156	1.36	1.23*E* − 02	0.48
L-homoserine	14.19	218	1.14	2.34*E* − 02	−0.25
L-sorbose	23.15	103	1.62	5.04*E* − 04	−0.44
L-tryptophan	28.60	202	1.76	1.09*E* − 03	−0.58
Lyxose	6.02	107	1.20	2.21*E* − 02	−0.09
Maleimide	7.36	154	1.37	8.22*E* − 03	−0.29
Methanephosphonothioic acid	16.06	241	1.61	5.63*E* − 03	−0.41
Montanic acid	31.25	369	2.03	1.17*E* − 04	−0.61
Morpholine	5.83	89	1.21	4.24*E* − 02	−0.24
Myo-inositol	20.15	305	1.54	3.55*E* − 03	−0.43
N,n-dimethyl-4-nitroso-aniline	19.58	207	2.03	1.31*E* − 04	−1.23
N-acetyl-d-mannosamine	17.27	260	1.63	2.04*E* − 03	0.21
N-acetylornithine	15.05	174	2.19	3.11*E* − 07	0.65
N-acetylputrescine	13.23	174	1.75	1.10*E* − 03	0.14
Nicotianamine	12.76	240	1.47	4.44*E* − 03	−0.18
Nonadecanoic acid	30.27	355	1.35	2.53*E* − 02	−0.28
Oleamide	29.27	131	1.37	2.35*E* − 02	0.08
O-phosphoethanolamine	21.28	299	1.51	2.48*E* − 02	0.70
O-propyl isopropylphosphonate	10.76	181	1.92	1.37*E* − 04	0.50
Palmitelaidic acid	26.08	117	1.04	3.70*E* − 02	0.44
Phenylalanine	16.27	120	1.42	9.91*E* − 03	−0.28
P-hydroxylphenyllactic acid	23.35	331	1.62	3.80*E* − 04	1.13
Piceatannol	22.92	295	1.98	1.40*E* − 05	−0.60
Proline	11.02	142	1.19	2.54*E* − 02	−0.29
Putrescine	13.33	174	1.83	4.05*E* − 04	0.12
Pyrophosphate	18.72	451	1.91	5.02*E* − 05	0.95
Pyrrole-2-carboxylic acid	11.33	240	1.75	7.15*E* − 04	−0.22
Pyruvic acid	6.60	174	1.66	2.76*E* − 04	−0.35
Quinic acid	19.59	345	2.00	6.19*E* − 04	−0.99
Resorcinol	7.33	225	1.05	4.04*E* − 02	−0.10
Resveratrol	11.86	341	1.55	4.35*E* − 03	−0.24
Ribose-5-phosphate	11.55	180	1.16	6.81*E* − 04	−0.68
Scyllo-inositol	26.13	318	1.19	1.80*E* − 02	−0.60
Sedoheptulose	27.75	319	1.27	4.00*E* − 02	−0.35
Sorbitol	24.41	319	1.40	7.55*E* − 03	−0.48
Stearic acid	29.19	117	1.52	5.26*E* − 03	−0.25
Tetracosane	10.67	85	1.34	1.60*E* − 02	0.04
Thymine	14.50	255	2.15	1.22*E* − 04	−1.41
Tocopherol acetate	20.77	430	1.85	2.47*E* − 04	−0.83
Trehalose-6-phosphate	30.29	315	1.02	4.34*E* − 02	−0.22
Trisaccharide	33.11	284	1.55	1.00*E* − 02	−2.14
Uracil	11.80	241	1.25	3.62*E* − 02	−0.39
Uridine 5'-monophosphate	20.50	262	1.69	2.51*E* − 03	−0.78
Xanthosine	33.02	325	1.02	4.37*E* − 02	−0.63
Xylofuranose	19.05	217	1.54	2.96*E* − 03	−0.51
Xylulose	19.12	263	1.59	2.78*E* − 03	−0.46

### Association Analysis of Microbial Diversity With Serum Metabolites

**FIGURE 6:**
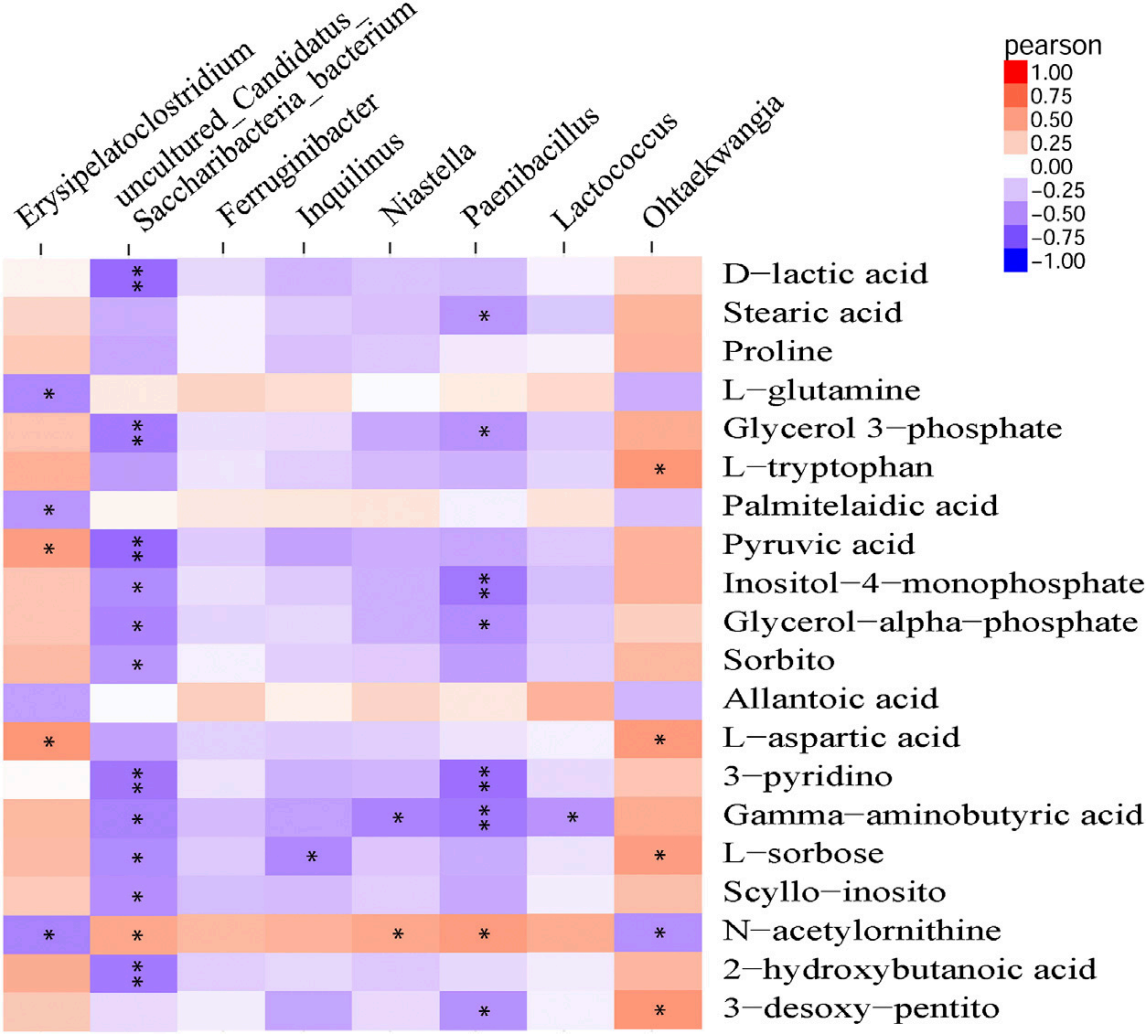
Pearson correlation analysis between modified metabolites and microbial genera by repeated oral administration of β-CD for 14 days *p < 0.05 and **p < 0.01 according to Pearson correlation.

Pearson correlation analysis was performed to detect correlations between gut microbiota and metabolite profiles modified by β-CD treatment. The phylum *Proteobacteria* was significantly negatively related to 3-pyridinol, nicotianamine, xanthosine and 2-deoxypentonic acid. At the genus level, *Ohtaekwangia* (mainly positive), *Paenibacillus* (mainly negative), *uncultured Candidatus Saccharibacteria bacterium* (mainly negative), and *Erysipelatoclostridium* (mainly positive), were closely related to many serum differential metabolites. However, the changes in *Ferruginibacter* were only significantly positively related to changes in putrescine (*p* < 0.05), and *Lactococcus* was significantly related to γ-aminobutyric acid (*p* < 0.05), *O*-phosphoethanolamine (*p* < 0.01), and bisphenol A (*p* < 0.01). The association analysis between the top 20 metabolites and microbial genera is shown in Figure 6, and the results at the levels of phylum, class, order, and family are shown in Supplementary Figure S5.

## Discussion

β-CD with a hydrophobicity cavity is popular in many fields, but the interaction between β-CD and organisms is little understood. In this study, the effects of oral exposure to β-CD on serum metabolites and gut microbiota were detected. A total of 1 phylum, 2 classes, 3 orders, 6 families and 8 genera were significantly different between the control and β-CDtreated groups. A total of 112 serum differential metabolites (89 downregulated and 23 upregulated) were detected after 14 days repeated oral administration of β-CD. Body weight as an important indicator for health was recorded, and no difference was found between the control and β-CD groups.

*Proteobacteria* contains several opportunistic pathogens and is suggested to exert proinflammatory activity both locally and systemically (Li et al., 2018; Ceccarani et al., 2020). The increased abundance of *Proteobacteria* induced by β-CD was significantly negatively related to serum differential metabolites of 3-pyridinol, nicotianamine, xanthosine and 2-deoxypentonic acid. Nicotianamine, a nonprotein amino acid, could inhibit the angiotensin-I-converting enzyme in the renin–angiotensin system and regulate blood pressure (Hayashi and Kimoto, 2007). Xanthosine is the intermediate in the metabolism of purine, and plays an important role in gene synthesis and metabolic regulation (Zhang et al., 2019). The effect of β-CD exposure on *Clostridiales vadinBB60 group* was highly correlated with urinary metabolites, and could also be changed by chronic cadmium exposure (He et al., 2020). The *Cystobacteraceae* family is well known for chitinase activity, and was upregulated by β-CD (Sharma and Subramanian, 2017). *Acidimicrobiaceae* is frequently encountered in acidic, metal-laden environments where they characteristically oxidize ferrous iron (Pinto et al., 2016). Fan et al. (2018) reported that *Acidimicrobiaceae* is capable of iron metabolism in the cyanobacteria-dominated open water zone. We speculated that the increased abundance of *Acidimicrobiaceae* induced by β-CD may affect iron metabolism in animals. *Paenibacillus* spp. upregulated by β-CD have been recognized as spoilage microorganisms and harmful to health, and they can survive conventional pasteurization regimens and grow during refrigerated storage (Nakano 2020). *Paenibacillus* was negatively related to many serum differential metabolites according to Pearson correlation analysis in our study. *Erysipelatoclostridium* is part of the normal gut microbiota but could become an opportunistic pathogen (Khan and Chousalkar, 2020). *Lactococci* are Gram-positive cocci and the *Lactococcus* genus includes five species, in which *Lactococcus lactis* is commonly used as a probiotic in food production and medical applications (Casalta and Montel, 2008; Rodrigues et al., 2016). β-CD-induced downregulation of *Erysipelatoclostridium* and upregulation of *Lactococcus* could be beneficial to the health of the host. Moreover, *Lactococcus* is significantly related to γ-aminobutyric acid, which is the principal inhibitory neurotransmitter and regulates neuronal excitability (Yi et al., 2017). *Ferruginibacter* upregulated by β-CD plays a key role in degrading organic components and denitrification (Liu et al., 2018). *Ferruginibacter* was only significantly positively related to the changed metabolites of putrescine, which is a naturally occurring polycation and plays an important role in cell growth, differentiation, and gene expression (Kwak et al., 2003). *Uncultured Candidatus Saccharibacteria bacterium* contributes to ammonium nitrogen (NH_4_^+^-N) removal (Ouyang et al., 2019), and was upregulated and negatively related to many changed serum metabolites in our study.

Pathways including ABC transporters, pyrimidine metabolism, purine metabolism, glucagon signaling, insulin signaling, glycolysis/gluconeogenesis and NAFLD were enriched by analysis of differential metabolites. ABC transporters mediate the ATP-dependent cellular export of a plethora of toxic metabolites and xenobiotic substances, and play key roles in multidrug resistance (Salustiano et al., 2020; Xiao et al., 2020). Dysregulation of the ABC transporters pathway indicates that animals might be under toxic stress after exposure to β-CD. It has also been reported that β-CD upregulates ABCA1 expression in vascular smooth muscle cells (Zhang C.-J. et al., 2020). Pyrimidines and purines are important components of DNA replication and RNA synthesis (Liu et al., 2019). Purines play a key role in neurotransmission and neuromodulation. Signaling molecules such as ATP and adenosine all belong to the purinergic system (Wang et al., 2019). Aberrations in purine metabolism by β-CD can affect neurological function and cell signal transduction. Abnormality of pyrimidine metabolism induced by β-CD may increase the risk of Alzheimer’s disease, immunodeficiency, growth retardation and aging (Wan et al., 2019). Glucagon promotes glycogenolysis and gluconeogenesis to elevate blood glucose levels in response to fasting, while insulin has the opposite effect (Jia et al., 2020; Liu et al., 2020). The glucagon signaling pathway, glycolysis/gluconeogenesis and insulin signaling pathway, as well as serum glucose level, were disturbed in this study. Therefore, we inferred that β-CD, a cyclic oligosaccharide consisting of seven glucose units (Alonso et al., 2018), could affect glucose metabolism after repeated oral administration. A previous study reported that intermediates of the glycolysis/gluconeogenesis pathway were related to insulin resistance, prediabetes, and diabetes (Guasch-Ferre et al., 2020). Diabetes and insulin resistance are both risk factors related to NAFLD (Komazaki et al., 2017). Path analysis showed that β-CD exposure also increased the risk of NAFLD in our study.

## Conclusion

In this study, the effects of oral exposure to β-CD on serum metabolites and gut microbiota were investigated in mice. Body weight or coefficients of organs were not changed after 14 days repeated oral administration. β-CD did not significantly affect the α-diversity indexes, but disturbed the structure of the gut microbiota according to PCA. The regulated genera of *Ohtaekwangia*, *Paenibacillus*, *uncultured Candidatus Saccharibacteria bacterium*, and *Erysipelatoclostridium* were closely related to many serum differential metabolites according to Pearson correlation analysis. The metabolic pathway of ABC transporters, pyrimidine metabolism, purine metabolism, glucagon signaling pathway, and insulin signaling pathway were enriched by KEGG pathway analysis. Our study revealed the effects of β-CD on gut microbiota, serum metabolites and their correlation, which should be considered before their application.

## Data Availability Statement

The 16s sequencing data has been deposited into BioProject (accession: PRJNA675411).

## Ethics Statement

The animal study was reviewed and approved by The Committee of Medical Ethics and Welfare for Experimental Animals, Henan University School of Medicine, China.

## Author Contributions

YY and XW developed the idea and designed the research. SL and XZ performed the experiments. SL, YF, QJ, and CN analyzed the data. SL wrote the draft of the manuscript. YY and XW contributed to revise the writing. All authors read and approved the submitted version.

## Funding

This work was supported by research funds from the National Natural Science Foundation of China (Grant No. 81971280, and No. 81600974), the Key Science and Technology Program of Henan Province in China (Grant No. 192102310151, No. 202102310213, and No. 192102310080), the Program for Young Key Teacher of Henan Province (Grant No. 2020GGJS037), and the Youth Talent Promotion Plan of Henan Association for Science and Technology (Grant No. 2020HYTP054).

## Conflict of Interest

The authors declare that the research was conducted in the absence of any commercial or financial relationships that could be construed as a potential conflict of interest.
