# Supplementary material for: Gut Microbiota Combined With Metabolomics Reveals the Repeated Dose Oral Toxicity of β-Cyclodextrin in Mice
Source: Front Pharmacol. 2021 Jan 14;11:574607. doi: 10.3389/fphar.2020.574607 (PMC7845417; doi:10.3389/fphar.2020.574607)
Supplement: Supplementary file 1 [file datasheet1.docx]

**Supplementary materials**

**
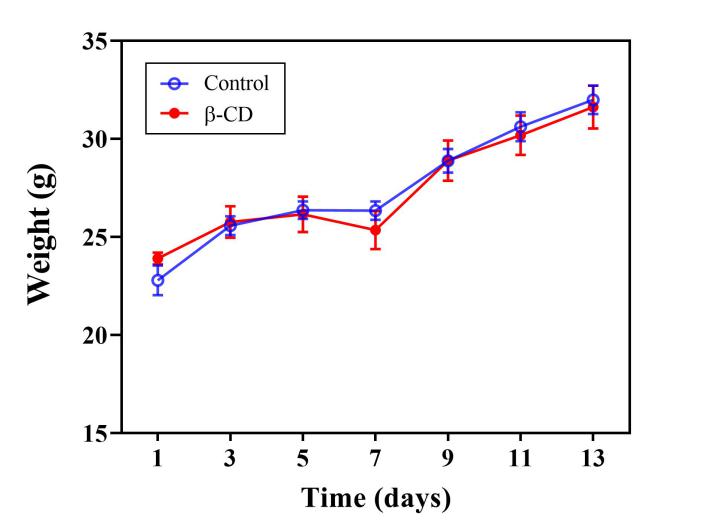
**

**Figure S1.** Body weight of mice during repeated oral administration of β-CD. All data are presented as mean ± SEM (n = 10).


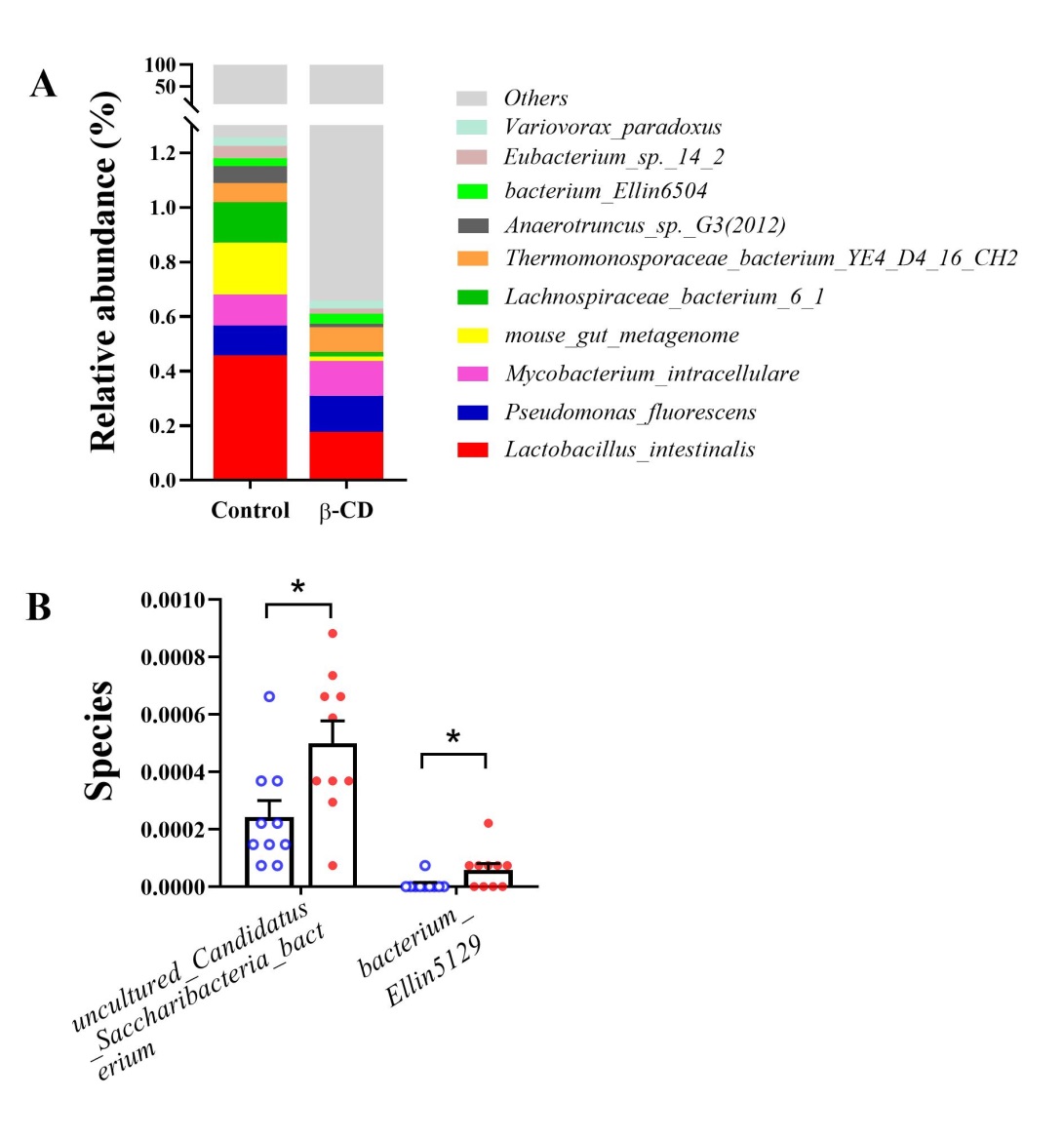


**Figure S2.** Taxonomic distributions (A) and significant differences (B) of bacteria at the species. **p* < 0.05 according to ANOVA (n = 10).

**A**

Control

**B**

β−CD

**C**

**Figure S3.** Representative total ion current (TIC) chromatographs from A) control, B) β−CD, and C) QC group.


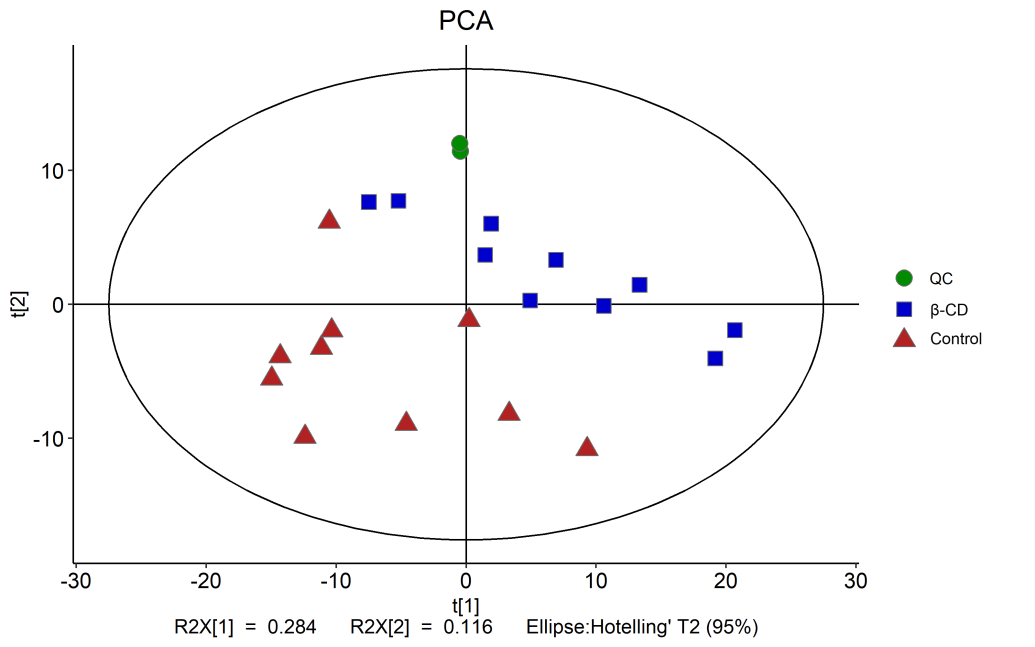


**Figure S4.** Score plots of the PCA model of all serum samples and QC samples.


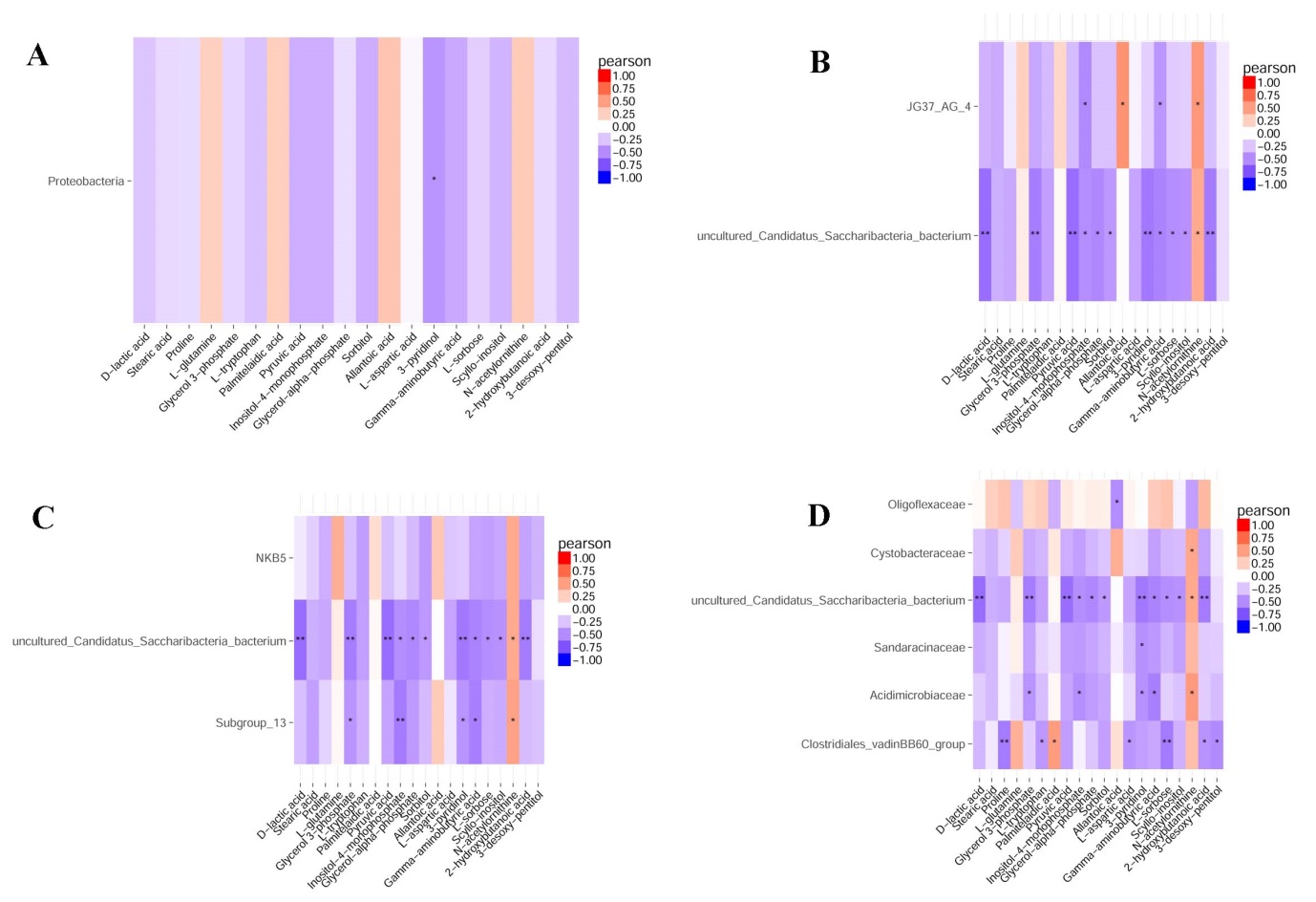


**Figure S5.** Pearson correlation analysis between modified metabolites and gut microbiota by repeated oral administration of β-CD for 14 days. A) phylum, B) Class, C) Order, D) Family. *p < 0.05 and **p < 0.01 according to Pearson correlation.

**Table S1** Coefficients of organs to body weight (BW) of mice after repeated oral administration of β-CD

| Index | Control | β-CD |
| --- | --- | --- |
| Body Weight (g) | 31.01 ± 0.87 | 29.63 ± 1.31 |
| Liver (mg/g) | 52.93 ± 1.67 | 52.34 ± 1.26 |
| Lung (mg/g) | 6.83 ± 0.21 | 6.81 ± 0.22 |
| Spleen (mg/g) | 2.63 ± 0.23 | 2.92 ± 0.50 |
| Kidney (mg/g) | 13.78 ± 0.34 | 13.57 ± 0.26 |
| Heart (mg/g) | 5.42 ± 0.23 | 5.82 ± 0.20 |

**Table S2** Summary of the sample tags and OTUs

| Sample | clean tags | valid tags | valid percent | OTU counts |
| --- | --- | --- | --- | --- |
| Cont1 | 30911 | 23091 | 74.70% | 1241 |
| Cont2 | 39313 | 31973 | 81.33% | 1138 |
| Cont3 | 38078 | 29597 | 77.73% | 1257 |
| Cont4 | 30965 | 23093 | 74.58% | 1364 |
| Cont5 | 38635 | 29189 | 75.55% | 1312 |
| Cont6 | 41680 | 31964 | 76.69% | 1283 |
| Cont7 | 39877 | 29489 | 73.95% | 1403 |
| Cont8 | 41030 | 32955 | 80.32% | 1124 |
| Cont9 | 38644 | 31602 | 81.78% | 1064 |
| Cont10 | 39117 | 30526 | 78.04% | 1277 |
| β-CD1 | 38577 | 31548 | 81.78% | 1187 |
| β-CD2 | 25914 | 18499 | 71.39% | 1373 |
| β-CD3 | 40982 | 32707 | 79.81% | 1152 |
| β-CD4 | 40752 | 30131 | 73.94% | 1463 |
| β-CD5 | 39190 | 28976 | 73.94% | 1359 |
| β-CD6 | 41962 | 33139 | 78.97% | 1085 |
| β-CD7 | 32424 | 23569 | 72.69% | 1457 |
| β-CD8 | 39618 | 30718 | 77.54% | 1294 |
| β-CD9 | 33202 | 26004 | 78.32% | 1244 |
| β-CD10 | 22288 | 17004 | 76.29% | 1351 |
